# Supplementary figures and images for: Differential epigenetic regulation of glucose-induced alteration of miR-9 in retinal and cardiac endothelial cells
Source: PLoS One. 2026 May 21;21(5):e0349188. doi: 10.1371/journal.pone.0349188 (PMC13193401; doi:10.1371/journal.pone.0349188)

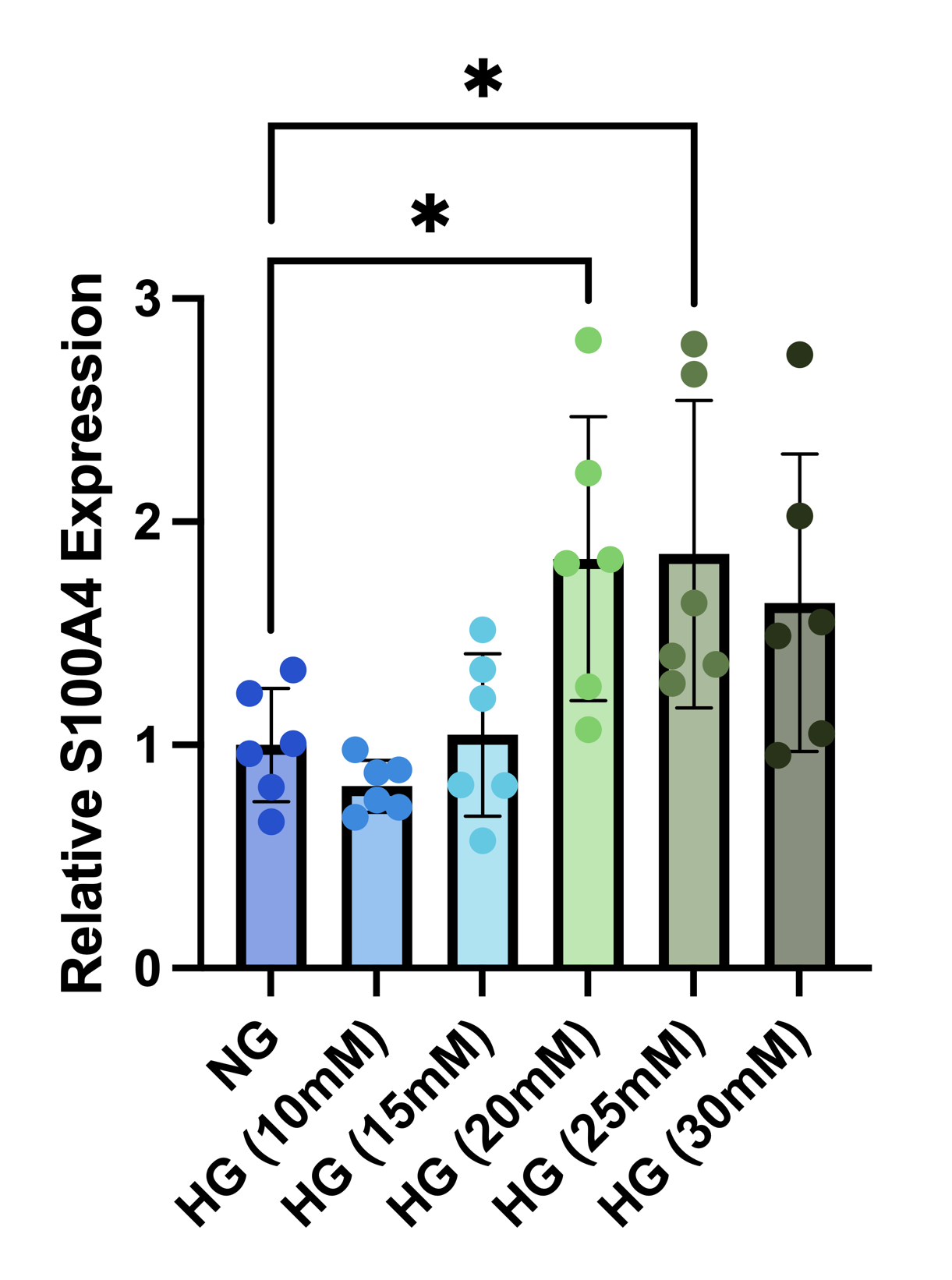

Supplement: S1 Fig — Various concentrations of high glucose were tested to confirm the optimal glucose concentration for gene expression changes without severe glucotoxicity. S100A4 was used as a marker for downstream changes. High glucose (HG) at 20 and 25 mM showed significant differences compared to normal glucose (NG; 5mM). [n = 6; RNA expression presented as ratio to β-actin mRNA and normalized to the NG group; data presented as mean ± SD; * = p < 0.05]. (TIFF) [file pone.0349188.s001.tiff]

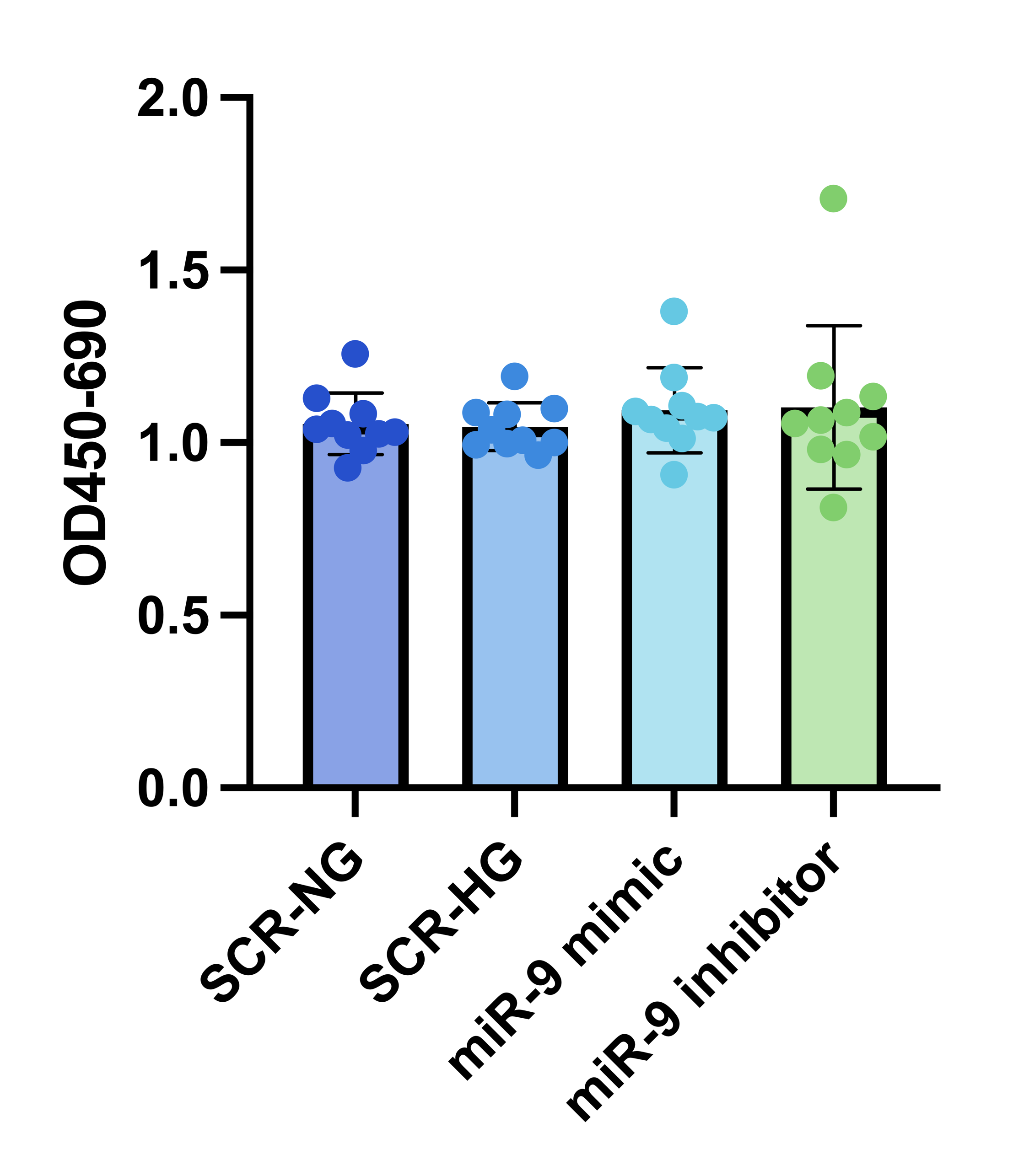

Supplement: S2 Fig — High glucose (HG; 25mM) treatment did not reduce cell viability in retinal MECs. miR-9 overexpression or inhibition also do not significantly alter the OD450 readings, meaning they do not directly influence cell viability. [n = 10; SCR = scrambled siRNA control; NG = normal glucose (5mM); data presented as mean ± SD; * = p < 0.05]. (TIFF) [file pone.0349188.s002.tiff]

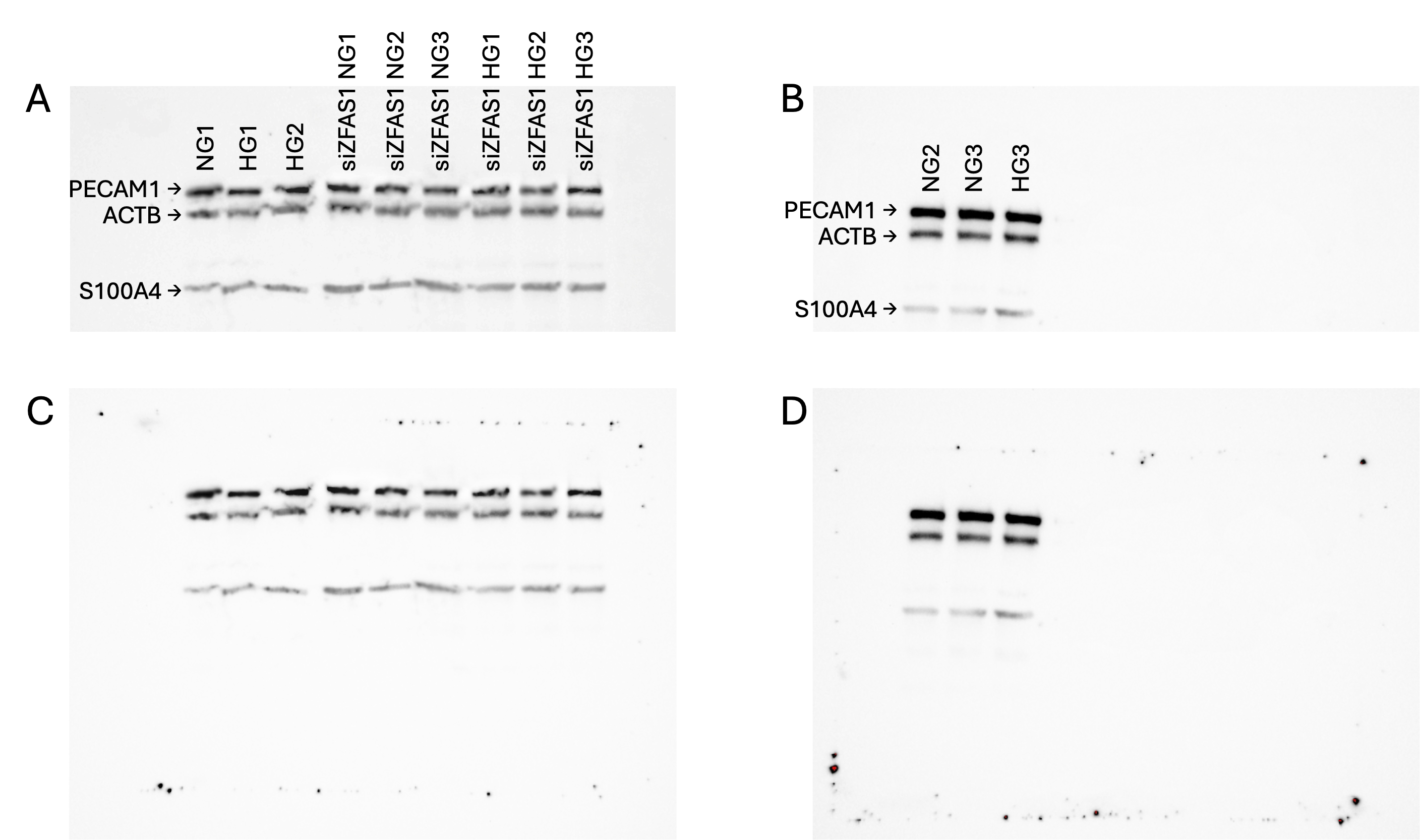

Supplement: S3 Fig — Annotated images of A) blot 1 and B) blot 2, representing one single experiment, performed simultaneously. Raw images of C) blot 1 and D) blot 2 are also provided. [SCR = scrambled siRNA control; NG = normal glucose (5 mM); HG = high glucose (25mM)]. (TIFF) [file pone.0349188.s003.tiff]
