# Supplementary material for: Differential epigenetic regulation of glucose-induced alteration of miR-9 in retinal and cardiac endothelial cells
Source: PLoS One. 2026 May 21;21(5):e0349188. doi: 10.1371/journal.pone.0349188 (PMC13193401; doi:10.1371/journal.pone.0349188)
Supplement: Attachment — Submitted filename: Response_to_reviewers_auresp_2.docx [file pone.0349188.s006.docx]

Reviewer #1: Thank you to the authors for the revision. The manuscript has improved significantly, especially with the addition of the ChIP data and protein validation. The study is now much clearer and better supported. I only have a few suggestions.
1. Please clearly state the number of biological replicates (n) for each experiment in the figure legends or method section. It would also help to clarify whether these were independent experiments.

We have added n values to each figure legend

2. For the ChIP experiments, please briefly clarify how enrichment was calculated (e.g., relative to IgG or input). A short clarification would improve transparency.

We had briefly mentioned of the enrichment calculation relative to IgG in the figure legend, but we have now added more information directly to the methods section.

3. Since DNMT1 recruitment is important in the retinal mechanism, it would be helpful to mention whether DNMT1 expression itself changes under high glucose (if data are available).

We have not quantified DNMT1 expression levels in the current study. However, we have previously (doi: 10.1167/iovs.62.3.20) that RNA expressions of DNMTs and PRC2 components are similarly upregulated under high glucose conditions. We have added comments to this effect in the discussion. [lines 338-339, and lines 348-350]

4. A small amount of editing in the discussion to simplify the explanation of the MALAT1–ZFAS1–miR-9 interaction would improve readability.

We have simplified the discussion surrounding the MALAT1-ZFAS1-miR-9 axis. [lines 379-427]
